# Post-transcriptional regulation of the human reduced folate carrier as a novel adaptive mechanism in response to folate excess or deficiency

**DOI:** 10.1042/BSR20140065

**Published:** 2014-08-06

**Authors:** Zhanjun Hou, Steve Orr, Larry H. Matherly

**Affiliations:** *Department of Oncology Wayne State University School of Medicine, Detroit, Michigan, U.S.A.; †Department of Pharmacology, Wayne State University School of Medicine, Detroit, Michigan, U.S.A.; ‡Molecular Therapeutics Programme, Barbara Ann Karmanos Cancer Institute, Detroit, Michigan, U.S.A.

**Keywords:** antifolate, folate, oligomerization, post-transcriptional regulation, reduced folate carrier, transporter, DAPI, 4′,6-diamidino-2-phenylindole, dihydrochloride, *dg*, deglycosylated, DSS, disuccinimidyl suberate, ER, endoplasmic reticulum, FR, folate receptor, hGAPDH, human glyceraldehyde-3-phosphate dehydrogenase, hRFC, human RFC, LCV, (6R,S)5-formyl tetrahydrofolate (leucovorin), Mtx, methotrexate, PCFT, proton-coupled folate transporter, PDI, protein disulfide isomerase, Pmx, pemetrexed, RFC, reduced folate carrier, sulfo-NHS-SS-biotin, sulfo-N-hydroxysuccinimide-SS-biotin, TMQ, trimetrexate (2,4-diamino-5-methyl-6-[(3,4,5-trimethoxyanilino)methyl]quinazoline, UTR, untranslated region, *wt*, wild-type

## Abstract

The RFC (reduced folate carrier) is the principal mechanism by which folates and clinically used antifolates are delivered to mammalian cells. hRFC (human RFC) is subject to complex transcriptional controls and exists as homo-oligomer. To explore the post-transcriptional regulation of hRFC by exogenous folates, hRFC-null HeLa cells were stably transfected with hRFC under control of a constitutive promoter. hRFC transcripts and the total membrane protein increased with increasing LCV [(6R,S)5-formyl tetrahydrofolate (leucovorin)] with a maximum at 20 nM LCV, attributable to reduced turnover of hRFC transcripts. hRFC homo-oligomerization was unaffected by increasing LCV. Cell surface hRFC paralleled [^3^H]methotrexate transport and increased from 0.5 to 2 nM LCV, and then decreased (~2-fold) with increasing LCV up to 20 nM. hRFC was localized to the cell surface at low LCV concentrations (0.5–1.5 nM). However, at higher LCV concentrations, significant intracellular hRFC was localized to the ER (endoplasmic reticulum), such that at 20 nM LCV, intracellular hRFC was predominated. Our results demonstrate a novel post-transcriptional regulation of hRFC involving: (i) increased hRFC transcripts and proteins, accompanying increased extracellular folates, attributable to differences in hRFC transcript stabilities; and (ii) increased retention of hRFC in the ER under conditions of folate excess, because of impaired intracellular trafficking and plasma membrane targeting.

## Abstract 2

A novel regulation of the physiologically/pharmacologically important human reduced folate carrier was demonstrated in response to increasing extracellular folates, involving: (i) increased transcripts and total protein, reflecting increased transcript stabilities; and (ii) increased endoplasmic reticulum trapping, due to impaired intracellular trafficking.

## INTRODUCTION

Reduced folate cofactors are essential one-carbon donors and acceptors in several metabolic reactions leading to nucleotides and critical amino acids [1]. Mammals cannot synthesize folates *de novo* and are absolutely dependent on folates obtained from exogenous dietary sources. The metabolic impact of folate deficiency can be severe and results in impaired synthesis of DNA, RNA, proteins and impaired biological methylation reactions [1,2]. Folate deficiency can manifest as a number of pathological states, including cancer [3,4].

Folates are hydrophilic anionic molecules that do not penetrate biological membranes by diffusion alone. Thus, cellular uptake of folate cofactors is essential to sustaining folate-dependent metabolism [5]. There are sophisticated membrane transport systems in mammals that facilitate folate uptake [6–8]. The ubiquitously expressed RFC (reduced folate carrier) (SLC19A1) is optimally active at neutral pH and is the major tissue membrane transporter for folate cofactors [9]. Other folate transport systems include the PCFT (proton-coupled folate transporter) that provides for intestinal folate absorption at the acidic pH characterizing the upper gastrointestinal tract [6,10], and FRα (folate receptor α) that internalizes folate cofactors across epithelial membranes by high affinity binding and endocytosis [7,11]. Members of the family of organic anion transporters (e.g., OAT2) transport a variety of organic anions including folates into tissues with low specificities and low affinities [5,12].

In addition to transporting folate cofactors, clinically relevant antifolate drugs used for cancer, including Mtx (methotrexate), Pmx (pemetrexed) and pralatrexate, are also substrates for these physiologically important folate uptake systems. For RFC, transport is a critical determinant of antitumour efficacy of these cytotoxic antifolates [9]. While recent reports described the development of novel 6-substituted pyrrolo[2,3-*d*]pyrimidine antifolates with selectivity for cellular uptake and tumour targeting by PCFT and FRs over RFC [13], RFC levels are nonetheless important determinants of antitumour activities for these agents, by impacting cellular folate pools, which compete for polyglutamylation and binding to intracellular enzyme targets [14].

The hRFC gene is located on chromosome 21q22.3 [15]. Five exons encode an hRFC protein consisting of 591 amino acids with 12 transmembrane domains [9]. hRFC is subject to intricate regulation of gene expression involving multiple promoters and non-coding exons [9]. With alternate splicing, there are up to 15 distinct 5′ UTRs (untranslated regions) fused to a common 1776 bp hRFC coding sequence [16,17]. Levels of hRFC transcripts and proteins among different tissues probably reflect the levels of both ubiquitous and tissue-specific transcription factors. hRFC transcripts with differing 5′UTRs showed striking differences in transcript stabilities, providing another means of regulating hRFC levels and membrane transport [18]. Post-translational modifications to the hRFC protein have not been extensively characterized with the exception of its *N*-glycosylation at Asn^58^ which has a nominal impact on hRFC trafficking and transport [19]. Finally, hRFC forms homo-oligomers [20]. Although each hRFC monomer functions as an independent transport unit, oligomerization appears to be critical to hRFC trafficking from the ER (endoplasmic reticulum) to the plasma membrane, thus providing another potential level of regulation [20,21].

Given the importance of RFC to *in vivo* folate homoeostasis and the impact of folate deficiency on human health and disease [9], interest in RFC regulation in relation to exogenous folate levels remains high. In mice-fed folate-deficient diets, RFC transcripts and proteins were elevated in small intestine but not in kidney [22]. In other studies, RFC levels were measured in response to changes in extracellular folate concentrations *in vitro*. For instance, substantially elevated RFC and membrane transport were induced in leukaemia cell lines (L1210, K562 and CCRF–CEM) selected for growth in sub-physiologic concentrations of folates [23–25], and at least part of the increased transport decreased upon addition of higher folate concentrations [24]. Likewise, in Caco-2 and HuTu-80 cells, hRFC transcripts and proteins were induced in response to folate deficiency and a putative folate-responsive transcriptionally active region was identified upstream of the hRFC-B minimal promoter [26]. However, in another study using transport up-regulated CEM/7A T-cell leukaemia cells and MCF7/MR breast cancer cells, hRFC levels decreased in response to folate deficiency [27]. This was suggested to be an adaptive response to folate deficiency designed to counteract the detrimental effects of high-affinity folate extrusion by hRFC.

To clarify the potential post-transcriptional mechanisms for regulating hRFC by folates, in this report, we performed systematic analyses of the relationships between extracellular folate concentrations and post-transcriptional impacts on the levels of hRFC transcripts and proteins, and on hRFC homo-oligomerization and intracellular trafficking, resulting in functional carrier at the plasma membrane surface. Our results demonstrate a novel adaptive regulation of hRFC in response to increasing extracellular folates involving: (i) increased hRFC transcripts and total hRFC proteins, reflecting changes in hRFC transcript stabilities; and (ii) increased ER-trapped hRFC, because of impaired intracellular trafficking and plasma membrane targeting. Our results are likely to be highly significant to *in vivo* folate homoeostasis and to the therapeutic effects of various antifolates in the face of changes in levels of exogenous folates.

## MATERIALS AND METHODS

### Reagents

[3′,5′,7-^3^H]Mtx (20 Ci/mmol) and [3′,5′,7,9-^3^H(*N*)](6*S*)-5-formyl tetrahydrofolate (16.6 Ci/mmol) were purchased from Moravek Biochemicals. Unlabelled Mtx and trimetrexate (glucuronate salt) [TMQ (trimetrexate (2,4-diamino-5-methyl-6-[(3,4,5-trimethoxyanilino)methyl]quinazoline)] were provided by the Drug Development Branch, NCI, National Institutes of Health (Bethesda, MD). Unlabelled LCV [(6*R*,*S*)5-formyl tetrahydrofolate (leucovorin)] was purchased from Sigma Chemical Company. Tissue culture reagents and supplies were purchased from the assorted vendors with the exception of FBS, which was purchased from Hyclone Technologies. The cross-linking reagent, DSS (disuccinimidyl suberate), was purchased from Pierce. DAPI (4′,6-diamidino-2-phenylindole, dihydrochloride) was purchased from Invitrogen.

### Cell culture

*wt* (wild-type) HeLa cells and hRFC-null Mtx-resistant HeLa cells, designated R5 [28], were gifts of Dr I. David Goldman (Bronx, New York), and were maintained as previously reported [29]. hRFC constructs, including *wt* hRFC^Myc−his10^ (hereafter, *wt*hRFC^Myc−his10^) and *dg* (deglycosylated) hRFC^Myc−his10^ (hereafter, *dg*hRFC^Myc−his10^), in which Asn^58^ is mutated to Gln to abolish *N*-glycosylation [20], were transfected into R5 cells with Lipofectamine Plus reagent (Invitrogen) [29]. Stable transfectants were selected with 1 mg/ml G418. Individual clones were isolated, expanded and screened on Western blots with Myc-specific antibody (see below). Clones expressing elevated *wt*hRFC^Myc−his10^ (hereafter, designated *wt*hRFC^Myc−his10^/R5) or *dg*hRFC^Myc−his10^ (*dg*hRFC^Myc−his10^/R5) were selected for further study and were maintained in G418 (100 μg/ml). For studies of the impact of exogenous folates on hRFC, folate cofactors were previously depleted for ~10 days by cell maintenance in folate-free RPMI 1640 containing 10% (v/v) dialysed FBS, penicillin (100 units/ml)/streptomycin (100 μg/ml) and 2 mM L-glutamine (hereafter referred to as ‘complete folate-free RPMI 1640 medium’); for the *wt*hRFC^Myc−his10^/R5 and *dg*hRFC^Myc−his10^/R5 cells, G418 (100 μg/ml) was included. Thymidine (10 μM) and adenosine (100 μM) were also included to circumvent folate requirements for nucleotide biosynthesis. For particular experiments, folate-depleted cells were washed in folate-free media, and cultured in complete folate-free RPMI 1640 without nucleosides and in the presence of LCV (0.5–200 nM). For experiments measuring LCV growth requirements for *wt*hRFC^Myc−his10^/R5 and *dg*hRFC^Myc−his10^/R5 cells, folate-depleted cells were cultured for 96 h with various concentrations of LCV; cell numbers were manually counted with a hemacytometer.

### Membrane transport experiments

For routine transport assays of hRFC, the cellular uptake of [^3^H]Mtx (0.5 μM) was measured over 2 min at 37°C in 60 mm dishes in Hepes-sucrose-Mg^+2^ buffer (20 mM Hepes, 235 mM sucrose, pH adjusted to 7.3 with MgO), as described previously [29]. Levels of intracellular radioactivity were expressed as pmol/mg of protein, calculated from direct measurements of radioactivity and protein contents [30] of the cell homogenates.

### Accumulation of [^3^H](6*S*) 5-formyl tetrahydrofolate

The *wt*hRFC^Myc−his10^/R5 and *dg*hRFC^Myc−his10^/R5 sublines were depleted of cellular folates by culturing in a complete folate-free RPMI 1640 medium with dialysed FBS, supplemented with 10 μM thymidine and 100 μM adenosine (above), and then treated with 0.5–200 nM of [^3^H]LCV [in these experiments, trace amounts of [^3^H](6*S*)5-formyl tetrahydrofolate were diluted with non-radioactive LCV, such that the actual concentration of (6*S*)5-formyl tetrahydrofolate including the radiolabelled folate form was one-half]. After 4 days, cells were washed with Dulbecco's PBS (3×). Cellular proteins were solubilized in 0.5 N NaOH and quantified using the Folin-phenol reagent [30]. Total cellular [^3^H]LCV accumulations were expressed as pmol/mg of protein, calculated from the direct measurements of radioactivity and protein contents of the cell homogenates.

### Preparations of crude membranes and Western blot analysis

Crude membranes, including both plasma and intracellular (e.g., ER) membrane fractions, were prepared by sonication, followed by differential centrifugation, as reported previously [29]. Proteins were solubilized in 10 mM Tris–HCl/1% (w/v) SDS with proteolytic inhibitors (Roche Applied Science) and analysed by SDS–PAGE on Laemmli gels [31], using 7.5% or gradient (4–20%) gels (Invitrogen), and then eletrotransferred to polyvinylidene difluoride membranes (Pierce) [29]. Detection and quantitation of immunoreactive proteins used anti-Myc antibody (Covance) and IRDye800-conjugated secondary antibody (LI-COR) with an Odyssey® infrared imaging system (LI-COR). To quantitate the hRFC forms (including the broadly-banding glycosylated hRFC forms and the 65 kDa deglycosylated hRFC), densitometry was performed using the Odyssey® (v 3.0) software. Linearity was confirmed over a 20-fold range of hRFC expression.

### Surface labelling with sulfo-NHS-SS-biotin (sulfo-N-hydroxysuccinimide-SS-biotin)

The Cell Surface Labelling Accessory Pack (Thermo Scientific) was used to biotinylate and isolate cell surface proteins so as to distinguish plasma membrane hRFC from hRFC that may be ‘trapped’ intracellularly. Briefly, cells were incubated with 0.25 mg/ml sulfo-NHS-SS-biotin in PBS for 30 min at 4°C, and then solubilized with lysis buffer. The lysates were centrifuged to remove the insoluble fraction and the supernatants were incubated with immobilized NeutrAvidin™ gel slurry for 1 h at room temperature. The beads were then washed three times with wash buffer containing protease inhibitors (Roche Applied Science). The bound proteins were eluted with 1× SDS–PAGE sample buffer [31] containing 50 mM dithiothreitol and analysed by SDS–PAGE/Western blotting.

### Cross-linking experiments

To detect total hRFC oligomers, cross-linking of *dg*hRFC^Myc−his10^/R5 with DSS (11.4 Å, flexible) was performed. Briefly, *dg*hRFC^Myc−his10^/R5 cells were washed with Dulbecco's PBS twice and then treated with DSS (in DMSO) at a final concentration of 0.5 mM for 30 min at room temperature. An equivalent amount of DMSO *in lieu* of DSS was added to an aliquot of cells as a negative control. The reactions were terminated by the addition of 20 mM Tris–HCl, pH7.5, and the cells were washed with PBS (2×). Cell pellets were frozen and stored at −20°C. Crude membranes were prepared by differential centrifugation [29]; proteins were solubilized and samples (20 μg) were analysed by SDS–PAGE and Western blotting (above).

### Real-time RT–PCR analysis of RFC transcripts

RNAs were prepared from *wt*hRFC^Myc−his10^/R5 or *dg*hRFC^Myc−his10^/R5 cells using TRIzol® reagent (Invitrogen). cDNAs were synthesized using random hexamers, RNase inhibitor, and MuLV reverse transcriptase (Invitrogen) and purified with the QIAquick® PCR Purification Kit (QIAGEN). Quantitative real-time RT–PCR was performed on a Roche LightCycler 1.2 (Roche Diagnostics) with gene-specific primers and FastStart® DNA Master SYBR Green I Reaction Mix (Roche Applied Science) [32]. Transcript levels for hRFC were normalized to those for hGAPDH (human glyceraldehyde-3-phosphate dehydrogenase). Primers and PCR conditions were identical to those previously reported [32]. External standard curves were constructed for hRFC and hGAPDH, using serial dilutions of linearized templates, prepared by amplification from suitable cDNA templates, subcloning into a TA cloning vector (PCR-Topo; Invitrogen) and restriction digestions. For transcript stability experiments, *dg*hRFC^Myc−his10^/R5 cells were treated with 10 μg/ml actinomycin D and RNAs were prepared at 2 h intervals over 10 h. hRFC transcripts were normalized to hGAPDH as hGAPDH transcript levels did not significantly change over 10 h in the presence of actinomycin D. Normalized hRFC transcripts levels were plotted on semi-logarithmic plots versus time for calculations of first-order transcript turnover rates and half-lives [18].

### Confocal microscopy

For confocal microscopy, stable R5 transfectants were plated in Lab-TekII® chamber slides (NalgeNunc International) with LCV treatments. After 96 h, the cells were fixed with 3.3% paraformaldehyde (in PBS), permeabilized with 0.1% (v/v) Triton X-100 (in PBS), and stained with primary antibodies, followed by incubation with secondary antibodies [33]. The primary antibodies used were mouse anti-Myc monoclonal antibody (Covance) and rabbit anti-PDI (protein disulfide isomerase; an ER marker) (Sigma) antibody. Fluorescent secondary antibodies were used, including Alexa Fluor®568 donkey anti-rabbit IgG and Alexa Fluor®488 donkey anti-mouse IgG (Molecular Probes). Cross-reactivities between primary antibodies and secondary antibodies were also tested. The slides were visualized with a Zeiss laser scanning microscope 510, using a 63× water immersion lens and the same parameter settings for all of the samples. Confocal analysis was performed at the Microscopy, Imaging and Cytometry Resources Core of the Barbara Ann Karmanos Cancer Institute.

### Statistical analysis

Statistical analyses were performed using GraphPad Prism v. 6.0.

## RESULTS

### Impact on *wt*hRFC of exogenous folate availability

In initial experiments, we measured *endogenous* hRFC transport activity in *wt* HeLa cells cultured in various concentrations of LCV. HeLa cells were previously depleted of endogenous folates by growth in complete folate-free media for 10 days (including 100 μM adenosine and 10 μM thymidine to circumvent folate requirements). Cells were then cultured without nucleosides and in the presence of 0.5–200 nM LCV for 96 h, after which initial rates of [^3^H]Mtx uptake were measured. Hela cells showed progressively decreased drug uptake with increasing LCV from the maximal level at 0.5 nM, decreasing approximately 2-fold by 200 nM LCV (Supplementary Figure S1; available at http://www.bioscirep.org/bsr/034/bsr034e130add.htm).

To isolate potential post-transcriptional (versus transcriptional) mechanisms for study that may account for the decreased RFC uptake, including relationships between extracellular folate concentrations and cell proliferation, hRFC levels, and transport function, hRFC-null R5 HeLa cells were stably transfected with *wt*hRFC^Myc−his10^ under control of a constitutive (CMV) promoter. Two stable clones (#10 and #21) were identified and showed nearly identical [^3^H]Mtx transport activity and hRFC protein expression (Supplementary Figure S2 at http://www.bioscirep.org/bsr/034/bsr034e130add.htm). Clone #10 (hereafter, designated *wt*hRFC^Myc−his10^/R5 for simplicity) was used for further studies.

**Figure 1:**
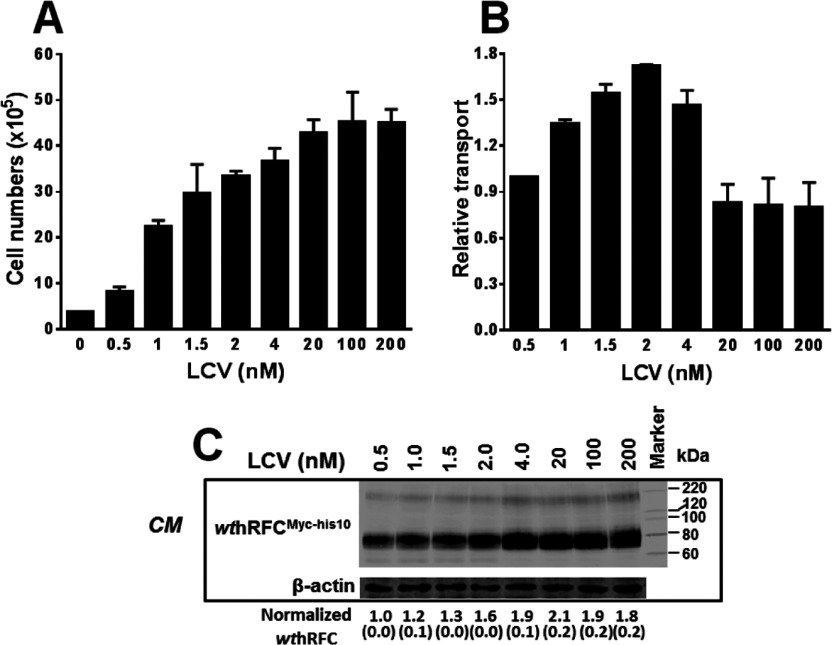
Characterization of *wt*hRFC^Myc−his10^/R5 cell responses to exogenous folate availability *wt*hRFC^Myc−his10^/R5 cells were depleted of endogenous folates by growth in complete folate-free media, in the presence of thymidine (10 μM) and adenosine (100 μM) for 10 days. Cells were then cultured in complete folate-free media in the presence of a range of LCV concentrations (0.5–200 nM) for 96 h, after which cell numbers, [^3^H]Mtx transport and hRFC protein levels were measured. (**A**) Growth of *wt*hRFC^Myc−his10^/R5 cells with increasing LCV up to 200 nM. Results are presented as mean values±range (error bars). The growth difference between 0 and 200 nM LCV was statistically significant (*P*<0.05). (**B**) *wt*hRFC^Myc−his10^/R5 cells were assayed for transport with [^3^H]Mtx (0.5 μM) for 2 min at 37°C. Transport results are normalized to that measured at a LCV concentration of 0.5 nM which was assigned a value of 1. Results are reported as mean values±range (error bars). The transport difference between 2 and 20 nM LCV was statistically significant (*P*<0.05). (**C**) hRFC protein levels are shown on a representative Western blot (two experiments were performed) of crude membrane proteins (15 μg) from *wt*hRFC^Myc−his10^/R5 cells cultured in the presence of increasing concentrations of LCV. The mean levels of normalized hRFC proteins (± S.E.) from two separate experiments, as determined by densitometry, are noted below each lane. Detection of immunoreactive *wt*hRFC^Myc−his10^ was with anti-Myc antibody and IRDye800-conjugated secondary antibody and used an Odyssey® Infrared Imaging System. *CM*, crude membranes. *wt*hRFC protein levels were normalized to levels of β-actin.

*wt*hRFC^Myc−his10^/R5 cells stably expressing *wt*hRFC^Myc−his10^ were again depleted of endogenous folates (above), then cultured without nucleosides and in the presence of LCV (0.5–200 nM) for 96 h, after which cell numbers, [^3^H]Mtx uptake and *wt*hRFC^Myc−his10^ protein levels were measured. *wt*hRFC^Myc−his10^/R5 cells showed a LCV concentration-dependent proliferation, with maximal growth at ~20 nM LCV which subsequently plateaued (Figure 1A). Relative [^3^H]Mtx uptake showed up to ~2-fold variations with increasing LCV, with a slightly increased drug uptake from 0.5 to 2 nM LCV, followed by a precipitous ~60% decrease from the peak level by 20 nM LCV that remained unchanged up to 200 nM LCV (Figure 1B). This pattern of hRFC transport function in response to extracellular concentrations of LCV was generally analogous to that observed with *wt* Hela cells (Supplementary Figure S1). Interestingly, *wt*hRFC^Myc−his10^ protein showed minimal relation to variations in transport activity, as there was a progressive increase in total *wt*hRFC^Myc−his10^ membrane protein (including both plasma membrane and ER fractions) from 0.5 to 20 nM LCV, and little-to-no further change in hRFC levels up to 200 nM LCV (Figure 1C). An identical pattern was seen for *wt*hRFC transcripts (results not shown).

As *wt*hRFC^Myc−his10^ in these experiments was expressed under control of a *constitutive* CMV promoter, our results imply a *post-transcriptional* modulation of hRFC levels and transport activity in response to the availability of extracellular folate cofactors. This effect appeared to be specific to hRFC, as human PCFT was not induced in response to extracellular folates in an analogous manner to that seen with hRFC (results not shown).

### Response of *dg*hRFC to variations in levels of extracellular folates

*wt*hRFC is extensively glycosylated which results in a relatively broadly migrating species on SDS–PAGE [25], which can complicate quantitative analyses of hRFC, including oligomeric hRFC forms [20]. To circumvent this, for additional studies and to further establish the generality of our initial findings with *wt*hRFC, we mutated Asn^58^ to Gln in *wt*hRFC^Myc−his10^ to abolish N-glycosylation. hRFC N-glycosylation has no significant effect on hRFC trafficking and transport [19]. The resulting *dg*hRFC^Myc−his10^ construct was transfected into R5 cells, and two stable clones (#6 and #11) were selected in G418 and assayed for hRFC expression and transport activity as before. *dg*hRFC^Myc−his10^ was detected as a sharp ~65 kDa band on SDS–PAGE and restored [^3^H]Mtx uptake over the very low level in hRFC-null R5 HeLa cells (Supplementary Figure S2).

**Figure 2:**
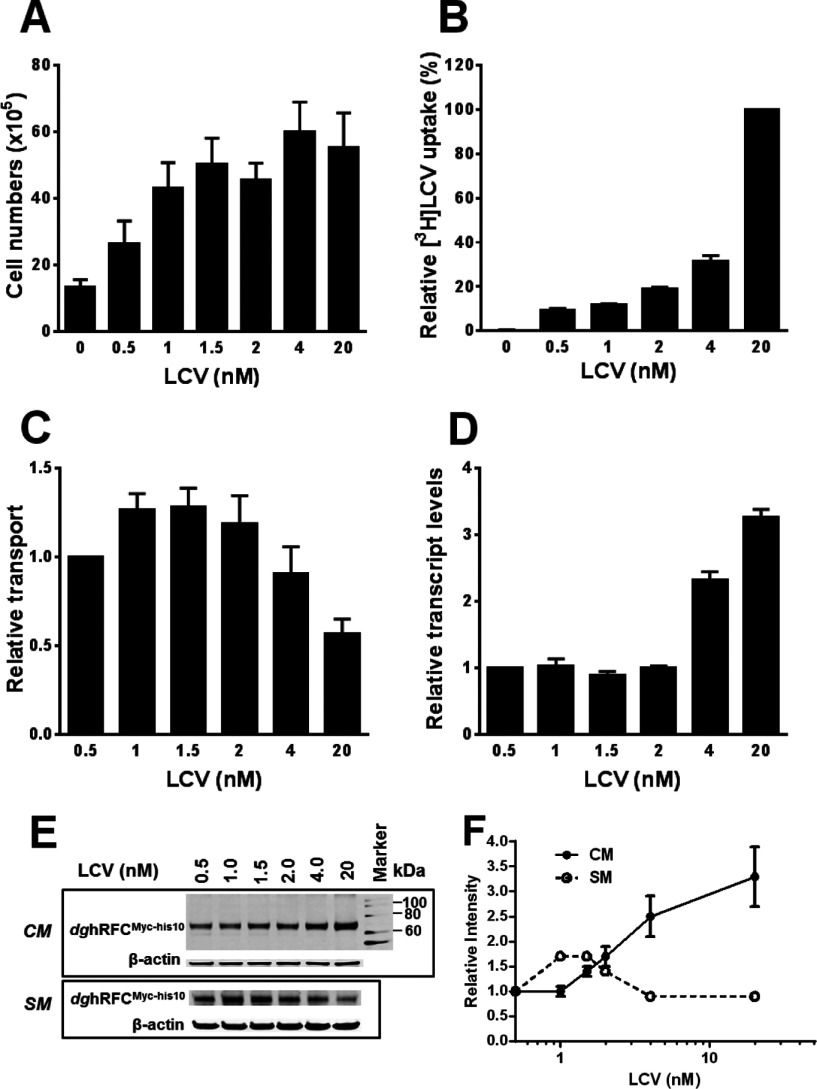
Characterization of *dg*hRFC^Myc−his10^/R5 cell responses to exogenous folate availability *dg*hRFC^Myc−his10^/R5 cells were depleted of endogenous folates by growth in complete folate-free media for 10 days. Cells were then cultured in the folate-free medium in the presence of a range of unlabelled LCV (0–20 nM) [for panels (**A**) and (**C**)–(**E**)] or [^3^H]LCV (0–20 nM) [for panel (**B**)] for 96 h. Cell numbers, [^3^H]LCV accumulations, [^3^H]Mtx transport, and hRFC protein levels (both in crude membranes and at the plasma membrane surface) were measured, as described in the Materials and Methods section. (**A**) LCV concentration dependence for *dg*hRFC^Myc−his10^/R5 cell proliferation is shown. Results are reported as mean values±S.E. (error bars) from four experiments. The growth difference between 0 and 20 nM LCV was statistically significant (*P*<0.05). (**B**) Experiments were performed with [^3^H]LCV to establish concentration-dependent accumulations of [^3^H]folates derived from [^3^H]LCV. [^3^H]LCV accumulations relative to that measured at 20 nM [^3^H]LCV (~4.3 pmole/mg) are presented as mean values±range (error bars). The uptake difference between 0.5 and 20 nM LCV was statistically significant (*P*<0.0001). (**C**) *dg*hRFC^Myc−his10^/R5 cells were assayed for transport with [^3^H]Mtx (0.5 μM) for 2 min at 37°C. Transport results were normalized to that measured at 0.5 nM LCV (assigned a value of 1) and are reported as mean values±S.E. (error bars) from five separate experiments. The transport difference between 1.5 and 20 nM LCV was statistically significant (*P*<0.05). (**D**) Quantitative real-time RT–PCR was performed for *dg*hRFC^Myc−his10^/R5 cells cultured with a range of LCV. Transcript levels for hRFC were normalized to those for hGAPDH. Results are expressed relative to those for a LCV concentration of 0.5 nM (assigned a value of 1) and are reported as mean values±S.E. (error bars) from seven experiments. The difference between transcript levels at 0.5 and 20 nM LCV was statistically significant (*P*<0.0001). (**E**) *dg*hRFC proteins are shown in a representative Western blot for crude membrane (*CM*) proteins, including both plasma membrane and intracellular (ER) membrane fractions (upper panel), and for surface membrane (*SM*) proteins (lower panel), from *dg*hRFC^Myc−his10^/R5 cells grown in the presence of varying concentrations of LCV. (**F**) The mean levels of normalized hRFC proteins (±S.E.) from three separate experiments, as determined by densitometry, for Western blots of *CM* and *SM* proteins in panel (**E**) are plotted. The differences in hRFC protein expression for the *CM* fraction between 0.5 and 20 nM LCV and for the *SM* fraction between 1 and 20 nM LCV were statistically significant (*P*<0.05). For the *SM* fraction, surface membrane proteins were labelled with sulfo-NHS-SS-biotin and isolated on immobilized NeutrAvidin™ gel. Detection of immunoreactive *dg*hRFC^Myc−his10^ was with anti-Myc antibody and IRDye800-conjugated secondary antibody with an Odyssey® Infrared Imaging System. β-Actin was used as a loading control. The molecular mass markers for SDS–PAGE are noted.

To further characterize the changes in hRFC levels and function in response to exogenous folates, clone #11 (hereafter, designated *dg*hRFC^Myc−his10^/R5) was depleted of endogenous folates as before, and then repleted in complete folate-free media with increasing LCV from 0.5 to 200 nM. After 96 h, cell densities were measured [results are shown in Figure 2(A) from 0.5 to 20 nM only, as there was no further impact of higher LCV concentrations in these experiments (results not shown)]. Parallel experiments were performed with [^3^H]LCV to establish concentration-dependent accumulations of [^3^H]folates. *dg*hRFC^Myc−his10^/R5 cells showed a similar LCV concentration dependence for cell proliferation to that seen with the *wt*hRFC transfectants (Figure 2A), and this was accompanied by concentration-dependent accumulation of [^3^H]folates from [^3^H]LCV (Figure 2B). By further analogy to *wt*hRFC, [^3^H]Mtx transport in *dg*hRFC^Myc−his10^/R5 cells increased at low LCV concentrations, with a maximum at 1–1.5 nM, followed by a ~55% decrease at 20 nM LCV (Figure 2C). There was no further decrease in transport activity up to 200 nM LCV (results not shown).

**Figure 3:**
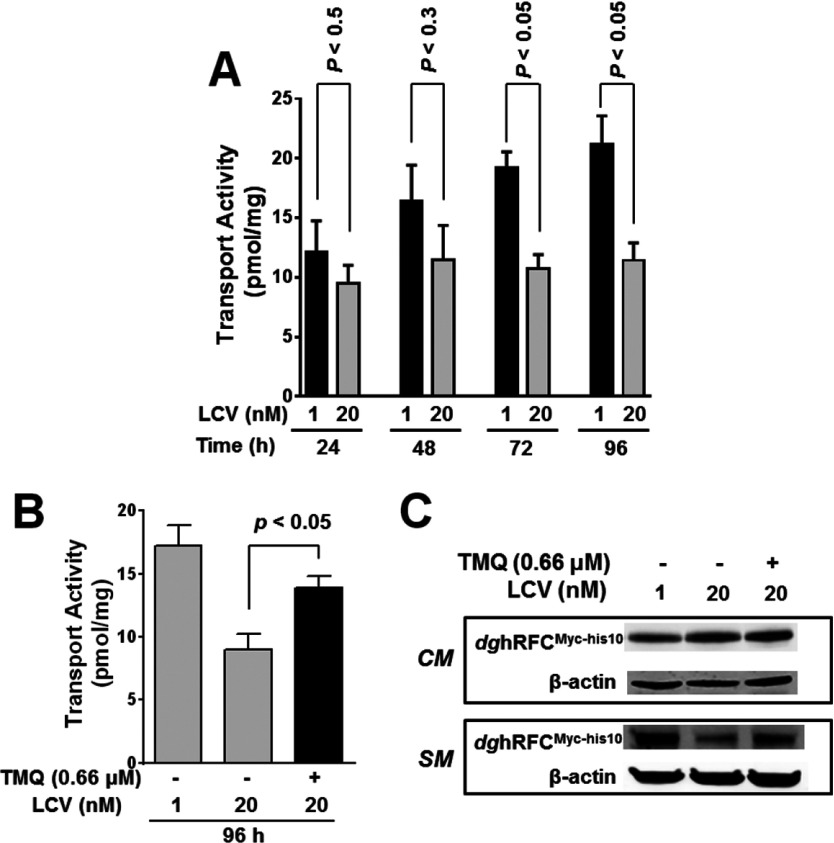
Dependence of *dg*hRFC responses to variations in LCV levels on time and folate metabolism (**A**) *dg*hRFC^Myc−his10^/R5 cells were depleted of cellular folates, and then cultured with 1 and 20 nM LCV for 24, 48, 72 and 96 h. Transport assays were performed at each time point with [^3^H]Mtx (0.5 μM) for 2 min at 37°C. Unpaired *t* tests were done for transport activity at two LCV levels at each time point with Prism (V. 6.0). (**B**, **C**) Cells were cultured with 1 and 20 nM LCV for 96 h with or without 0.66 μM TMQ, followed by transport assays (**B**) and Western blotting of *dg*hRFC^Myc−his10^ in crude membranes [includes both plasma membrane and intracellular (ER) membrane fractions] (*CM*) (**C**, upper panel)] and cell surface membranes (*SM*) (**C**, lower panel). For surface membrane proteins, cells were labelled with sulfo-NHS-SS-biotin, as described in the legend for Figure 2 and in the Materials and Methods section. In panel (**B**), the partial reversal of the effects of 20 nM LCV on hRFC membrane transport by TMQ was statistically significant (*P*<0.05). Results in panels (**A**) and (**B**) are for triplicate experiments (presented as mean values±S.E.). β-Actin was used as a loading control.

To establish the time dependence for the changes in *dg*hRFC^Myc−his10^ transport activity, we treated *dg*hRFC^Myc−his10^/R5 cells previously depleted of folates with 1 or 20 nM LCV from 24 to 96 h. As shown in Figure 3(A), for the cells treated with 1 nM LCV, there was a linear time-dependent increase in [^3^H]Mtx uptake that was maximal by 96 h, whereas the uptake was unchanged from 24 to 96 h for *dg*hRFC^Myc−his10^/R5 cells grown in 20 nM LCV.

As with cells expressing *wt*hRFC, the changes in hRFC transport activity in *dg*hRFC^Myc−his10^/R5 cells with increasing LCV were again accompanied by disparate increases in hRFC proteins in crude cell membranes, including both plasma membrane and ER fractions (Figure 2E, upper panel labelled ‘*CM*’ and Figure 2F). Furthermore, this was accompanied by parallel changes in hRFC transcripts (Figure 2D) (~2–3-fold increased when comparing hRFC transcripts and proteins between 1 and 20 nM LCV). However, when the *cell surface dg*hRFC^Myc−his10^ proteins were selectively biotinylated with membrane-impermeable sulfo-NHS-SS-biotin, then isolated on immobilized avidin, eluted and analysed on Western blots with anti-Myc antibody, the surface *dg*hRFC fraction initially *increased*, then *decreased* with increasing LCV in near exact proportion to the changes in [^3^H]Mtx transport [i.e., compare the Western blotting results for the cell surface *dg*hRFC^Myc−his10^ in Figure 2(E), lower panel (labelled ‘*SM*’) and densitometry measurements in Figure 2(F), to the transport data in Figure 2(C)].

The impact of LCV on [^3^H]Mtx uptake and cell surface (‘*SM*’) *dg*hRFC^Myc−his10^ proteins in these experiments appeared to depend on the metabolism of the exogenous LCV since this effect on hRFC was partly reversed (~35%) upon treatment with 20 nM LCV in the presence of TMQ (0.66 μM), a lipophilic antifolate that inhibits dihydrofolate reductase (Figures 3B and 3C, lower panel) [34]. However, the total *dg*hRFC protein in the crude membrane (‘*CM*’) fraction for cells cultured in 20 nM LCV was essentially unchanged accompanying TMQ treatment (Figure 3C, upper panel).

Collectively, these results suggest the existence of *post-transcriptional* regulatory mechanisms for hRFC that result in decreased surface hRFC and transport activity with increasing extracellular LCV, which, at least in part, depend on metabolism of (6*S*)5-formyl tetrahydrofolate.

### Transcript stabilities of *dg*hRFC in relation to folate availability

Total membrane *dg*hRFC^Myc−his10^ protein in *dg*hRFC^Myc−his10^/R5 cells treated with increasing LCV paralleled changes in hRFC transcripts (Figures 2D and 2E, upper panel, and Figure 2F). An analogous pattern was seen with *wt*hRFC, but not human PCFT (results not shown), both constitutively expressed under control of the same CMV promoter in the pCDNA3 vector in transporter-null HeLa cells. We reasoned that the elevated steady-state hRFC transcript levels with increasing LCV could best be rationalized in terms of differences in transcript stabilities. Although hRFC transcript stabilities were previously reported to regulate hRFC protein levels, attributable to distinct mRNA 5′UTRs [18], *dg*hRFC transcripts were all transcribed with the same 43 bp hRFC-B 5′UTR [17].

**Figure 4:**
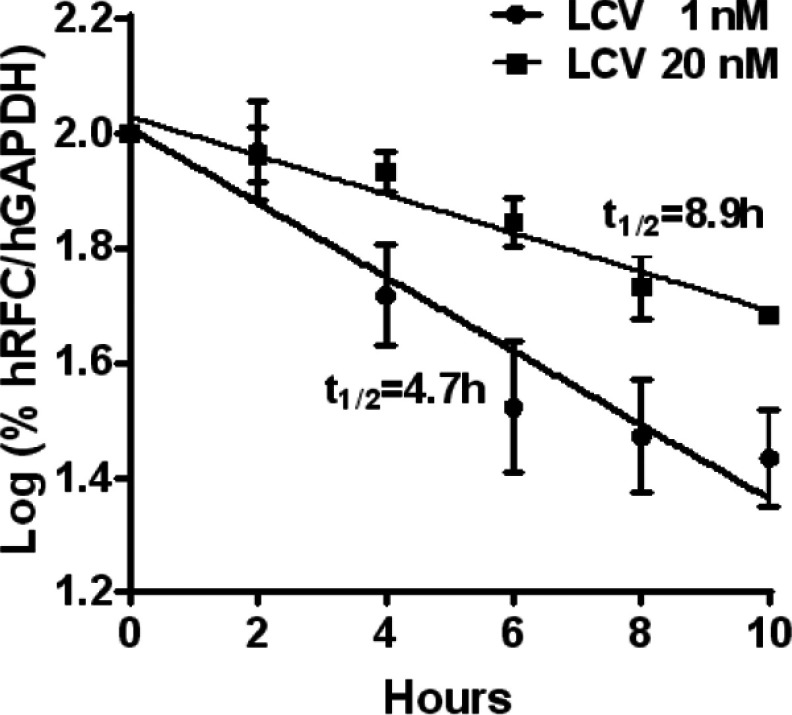
Transcript stabilities of *dg*hRFC in relation to folate availability Folate-depleted *dg*hRFC^Myc−his10^/R5 cells were cultured in 1 and 20 nM LCV for 96 h, then treated with actinomycin D (10 μg/ml) over 10 h. Total RNAs were isolated at 2 h intervals, following addition of actinomycin D, and hRFC transcripts were quantified by real-time PCR, normalized to hGAPDH. First-order decay rates and half-lives for relative hRFC transcript levels were calculated from semi-logarimic plots of relative hRFC transcript levels as a function of actinomycin D treatment times from two experiments (the data points shown are mean values±S.E.). hRFC transcript half-lives were calculated as 4.7 h for cells cultured in 1 nM LCV and as 8.9 h for cells cultured in 20 nM LCV.

To explore the impact of differences in extracellular LCV concentrations on *dg*hRFC transcript stabilities, we cultured folate-depleted *dg*hRFC^Myc−his10^/R5 cells in 1 and 20 nM LCV for 96 h, then treated the cells with actinomycin D (10 μg/ml) over 10 h. Total RNAs were isolated at 2 h intervals following actinomycin D treatments and hRFC transcripts were quantified by real-time PCR and normalized to hGAPDH transcripts. hGAPDH transcript levels did not change over 10 h in the presence of actinomycin D. First-order decay rates are shown in Figure 4 and half-lives of decay for relative hRFC transcript levels were calculated. hRFC transcripts were degraded at dramatically different rates for *dg*hRFC^Myc−his10^/R5 cells cultured in the different LCV concentrations. hRFC transcript half-lives were calculated as 8.9 h for cells cultured in 20 nM LCV, whereas for cells cultured in 1 nM LCV, the half-life was calculated as 4.7 h.

### Impact of changes in exogenous folate concentrations on *dg*hRFC oligomerization

**Figure 5:**
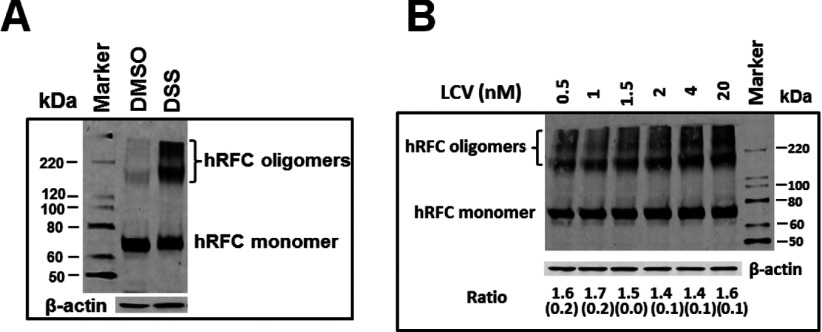
Impact of exogenous folate concentrations on *dg*hRFC oligomerization Folate-depleted *dg*hRFC^Myc−his10^/R5 cells were cultured over a range of LCV concentrations (0.5–20 nM), followed by treatment with DSS, an amine cross-linker that cross-links both surface and intracellular proteins. Following cross-linking, crude membrane fractions were prepared, solubilized with SDS and fractionated by SDS–PAGE (4–20% Tris–glycine gel) with detection by Western blotting with Myc-specific primary antibody and an IRDye800-conjugated secondary antibody and Odyssey® Infrared Imaging System. Substantial levels of higher molecular mass hRFC proteins were detected [approximating predicted masses of the hRFC trimer (~210 kDa) and tetramer (~280 kDa)] from cells treated with DSS, but not in untreated samples (panel **A**), following growth in 0.5–20 nM LCV. In a representative Western blot from triplicate experiments (panel **B**), oligomeric and monomeric *dg*hRFC^Myc−his10^ proteins were measured by DSS cross-linking from cells grown in varying LCV from 0.5 to 20 nM LCV. The mean ratios (±S.E.), as determined by densitometry, of total oligomeric to monomeric hRFC proteins are noted below each lane. Experimental details are provided in the Materials and Methods section.

To examine whether increasing levels of exogenous LCV can impact hRFC oligomerization, we treated folate-depleted *dg*hRFC^Myc−his10^/R5 cells cultured over a range of LCV concentrations (0.5–20 nM) with DSS, an amine cross-linker that cross-links both surface and intracellular hRFC proteins. Following cross-linking, crude membrane fractions were prepared, solubilized with SDS and fractionated by SDS–PAGE with detection by Western blotting with Myc-specific antibody. As shown in Figure 5(A), substantial levels of higher molecular mass hRFC proteins were detected [approximating masses of the hRFC trimeric (~210 kDa) and tetrameric (~280 kDa) forms] from cells treated with DSS but not in untreated (labelled ‘DMSO’) samples. Furthermore, whereas levels of oligomeric *dg*hRFC^Myc−his10^ progressively increased from 0.5 to 20 nM LCV, this was accompanied by similarly increased monomeric *dg*hRFC^Myc−his10^ such that the ratios of total oligomeric hRFC to hRFC monomers were unchanged (Figure 5B). These results strongly suggest that the formation of higher order *dg*hRFC^Myc−his10^ oligomeric species is not affected by folate depletion and resupplementation over a broad range of LCV concentrations.

### Effect of exogenous folates on intracellular trafficking of *dg*hRFC^Myc−his10^

The finding that both surface hRFC protein and transport activity decreased with increasing exogenous LCV, in spite of progressively elevated hRFC transcripts and total membrane (plasma and intracellular membranes) hRFC, implied that extracellular folate levels and the cellular folate status can affect hRFC trafficking and plasma membrane targeting and expression. As these effects occurred over a range of folate concentrations which span both normal and pathologic states, they are likely to be clinically relevant.

**Figure 6:**
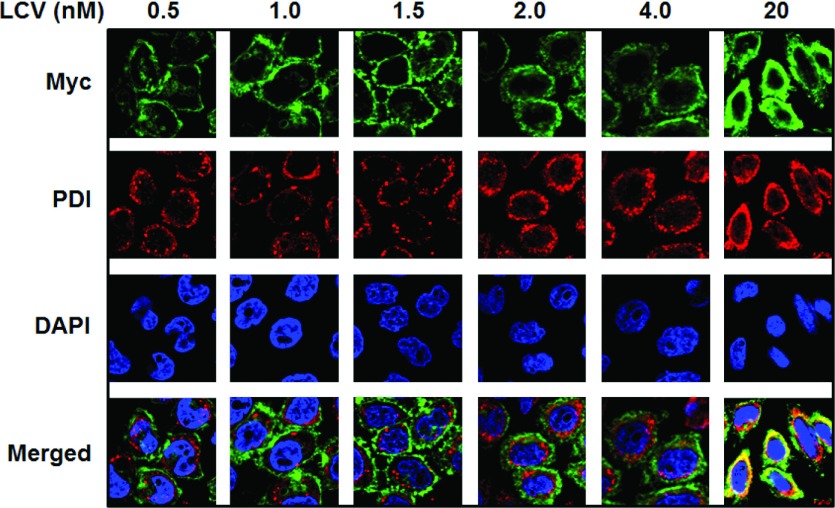
Effect of exogenous folates on intracellular trafficking of *dg*hRFC^Myc−his10^ Intracellular and surface *dg*hRFC^Myc−his10^ in *dg*hRFC^Myc−his10^/R5 cells (cultured in 0.5–20 nM LCV) were tracked by confocal microscopy and indirect immunofluorescence staining with mouse anti-Myc primary antibody and Alexa Fluor®488-conjugated anti-mouse secondary antibody. ER was stained with anti-PDI rabbit antibody and anti-rabbit (Alexa Fluor®568) secondary antibody, and nuclei were stained with DAPI. Individual and merged images are shown. Experimental details are provided in the Materials and Methods section.

We tracked intracellular and surface *dg*hRFC in *dg*hRFC^Myc−his10^/R5 cells cultured in 0.5–20 nM LCV by indirect immunofluorescence staining with mouse anti-Myc and Alexa Fluor®488 anti-mouse secondary antibodies and confocal microscopy. To simultaneously localize the ER with Alexa Fluor®488-stained *dg*hRFC^Myc−his10^, we used an ER membrane marker, PDI, with anti-PDI rabbit antibody and anti-rabbit (Alexa Fluor®568) secondary antibody. Nuclei were stained with DAPI. Individual and merged images are presented in Figure 6 for *dg*hRFC^Myc−his10^/R5 cells following folate depletion and repletion with LCV. As shown in Figure 6, at 0.5–1.5 nM LCV, *dg*hRFC^Myc−his10^ proteins were clearly localized at the plasma membrane surface, with no significant intracellular staining coincident with PDI. From 2–4 nM LCV, a major portion of the *dg*hRFC^Myc−his10^ was stained at the cell surface, with notable intracellular staining localized at the ER, as reflected in slight yellow staining in merged images. By 20 nM LCV, overall cell staining of *dg*hRFC^Myc−his10^ was substantially augmented with much of the *dg*hRFC^Myc−his10^ protein co-localized with PDI to the ER. Thus, increased extracellular LCV and intracellular folate cofactors appear to promote ‘trapping’ of newly synthesized hRFC in the ER, suggesting a novel mechanism for regulating hRFC in response to extracellular folate concentrations and providing an explanation for the disparate sulfo-NHS-SS-biotin surface labelling results with increasing LCV (Figures 2E and 2F).

## DISCUSSION

This report attempts to provide further clarity to role of exogenous folates in regulating hRFC, *independent* of possible effects on hRFC transcription. We document previously unrecognized *post-transcriptional* mechanisms which function under conditions that mimic changes in concentrations of extracellular folates, as might occur with *in vivo* folate deficiency or repletion. By ectopically expressing hRFC constructs in hRFC-null HeLa cells under control of a constitutive CMV promoter and culturing cells under well-defined conditions of folate deficiency or repletion, we were able to dissociate the effects of cellular folate status on steady-state hRFC transcripts and total cellular hRFC proteins from those involving hRFC transcription from endogenous promoters. We identified dual modes for regulating hRFC, including: (i) elevated hRFC transcripts and total hRFC membrane proteins which accompany growth in increased extracellular folates, attributable to enhanced hRFC transcript stability; and (ii) increased intracellular hRFC retention in the ER under conditions of folate excess, due to impaired intracellular trafficking and reduced plasma membrane surface targeting.

While the most of our studies were performed with *dg*hRFC^Myc−his10^, analogous results were obtained with a *wt*hRFC construct, and to some degree, endogenously ex-pressed *wt*hRFC in *wt* HeLa cells. Notably, our results with hRFC described herein are consistent with an earlier report in which [^3^H]Mtx influx was up-regulated in a CCRF–CEM cell line variant selected for growth in a sub-physiological level of LCV, but was substantially decreased by exposure to a higher more physiological concentration of LCV [24]. This effect was substantially reversed by treatment with TMQ [24]. Similarly, the treatment with 20 nM LCV in the presence of TMQ reversed the impact of LCV on [^3^H]Mtx uptake and cell surface *dg*hRFC^Myc−his10^ proteins in our experiments, suggesting a role for metabolism of the exogenous LCV.

Interestingly, the regulation of other folate transport systems was reported in response to changes in extracellular folates, analogous to those described here for hRFC. For instance, increased transcript levels were reported for FRα in KB human tumour cells cultured under folate-deficient conditions, because of both an increased transcription rate and a prolonged transcript half-life, resulting in elevated steady-state FRα mRNAs [35]. A mitoxantrone-resistant MCF-7/MR subline cultured in an elevated concentration of folate expressed high levels of the folate exporter ABCG2 (breast cancer resistant protein) in plasma membranes, whereas at a lower folate concentration, ABCG2 decreased and was primarily intracellular (as opposed to the plasma membrane), co-localizing with the ER-associated chaperone calnexin [36]. Collectively, these previous reports, combined with the results for hRFC described herein, suggest a broad-reaching regulatory adaptation of mammalian cells to conditions of folate depletion or excess, involving mechanisms critical to folate cofactor uptake and retention. These processes are likely to be highly significant to *in vivo* folate homoeostasis, and indeed may contribute to pathophysiologic conditions associated with folate deficiency and/or impact therapeutic efficacy of standard and targeted antifolates for cancer.

Studies of the mechanisms responsible for our findings with hRFC are underway. These include identification and localization of a putative folate-sensitive hRFC mRNA sequence(s) and protein factors that impact hRFC transcript stabilities, and identification of putative folate-responsive chaperones involved in folding of oligomeric hRFC proteins in the ER and in plasma membrane targeting of functional hRFC under conditions of maximal metabolic need. Indeed, sequestering nascent proteins from the degradation pathway by their interaction with ER-resident proteins such as calnexin, calreticulin, BiP or PDI will both protect these from the ER-associated degradation pathway and prevent secretion [37]. Clearly, the intricate balance between folding, aggregation and degradation of hRFC proteins in the ER in the presence of different levels of exogenous folates is an important topic needs to be explored further.

## Online data

Supplementary data
