# Supplementary material for: Post-transcriptional regulation of the human reduced folate carrier as a novel adaptive mechanism in response to folate excess or deficiency
Source: Biosci Rep. 2014 Aug 6;34(4):e00130. doi: 10.1042/BSR20140065 (PMC4122975; doi:10.1042/BSR20140065)
Supplement: Supplementary data [file bsr034e130add.pdf]

Biosci. Rep. (2014) / **34** / art:e00130 / doi 10.1042/BSR20140065

Original Paper

OPEN ACCESS

Bioscience Reports

www.bioscirep.org

Bioscience Reports

SUPPLEMENTARY DATA

Post-transcriptional regulation of the human reduced folate carrier as a novel adaptive mechanism in response to folate excess or deficiency

Zhanjun HOU*‡[1], Steve ORR*‡ and Larry H. MATHERLY*†‡

*Department of Oncology Wayne State University School of Medicine, Detroit, Michigan, U.S.A.
†Department of Pharmacology, Wayne State University School of Medicine, Detroit, Michigan, U.S.A.
‡Molecular Therapeutics Programme, Barbara Ann Karmanos Cancer Institute, Detroit, Michigan, U.S.A.

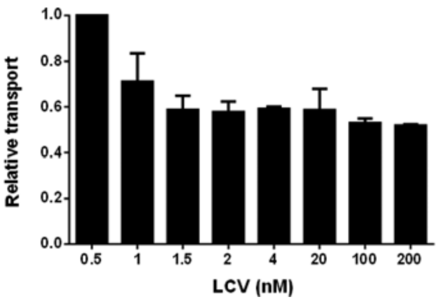

Figure S1 Characterization of Hela cell responses to exogenous folate availability
Hela cells were depleted of endogenous folates by growth in complete folate-free media, in the presence of thymidine (10 μM) and adenosine (100 μM) for 10 days. Cells were then cultured in complete folate-free media in the presence of a range of LCV concentrations (0.5–200 nM) for 96 h, and then assayed for transport with [^{3}H]Mtx (0.5 μM) for 2 min at 37°C. Transport results were normalized to transport measured at 0.5 nM LCV, which was assigned a value of 1. Results are reported as mean values ± standard errors (error bars) from three separate experiments. The transport differences measured at 0.5 and 200 nM LCV were statistically significant (P<0.05).

[1] To whom correspondence should be addressed (email houz@karmanos.org).

© 2014 The Author(s) This is an Open Access article distributed under the terms of the Creative Commons Attribution Licence (CC-BY) (http://creativecommons.org/licenses/by/3.0/) which permits unrestricted use, distribution and reproduction in any medium, provided the original work is properly cited.

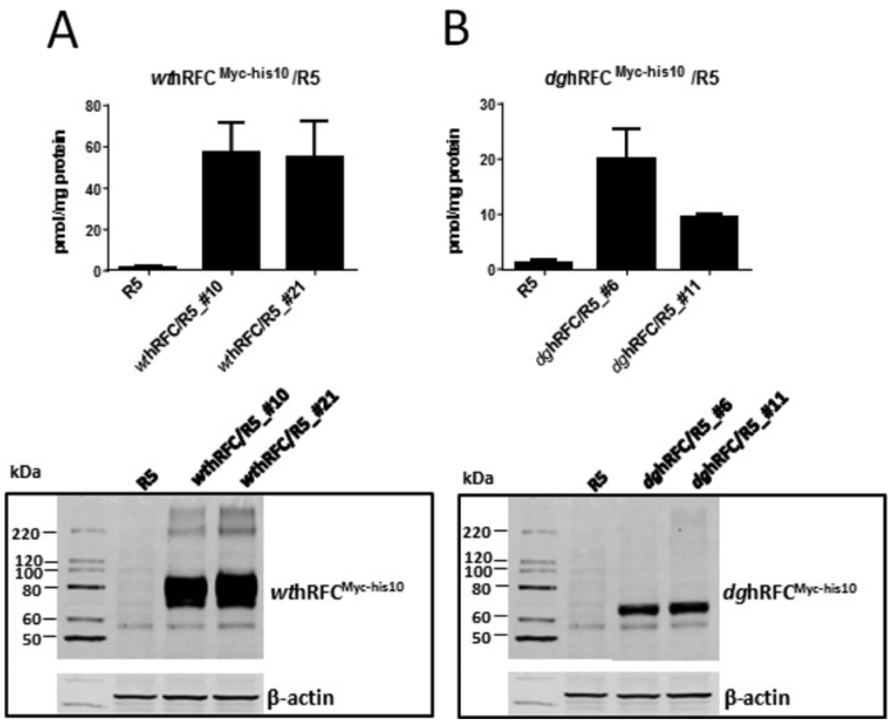

Figure S2 **Characterization of *wt* and *dg*hRFC$^{Myc-his10}$/R5 stable clones**
Both *wt* and *dg*hRFC$^{Myc-his10}$/R5 stable clones and RFC-null Hela derivative R5 cells were assayed for transport with [^{3}H]Mtx (0.5 μM) for 2 min at 37 °C. hRFC expression was analysed by Western blotting of crude membrane protein fractions. Detection of immunoreactive hRFC was with anti-Myc antibody and IRDye800-conjugated secondary antibody with an Odyssey® Infrared Imaging System. [^{3}H]Mtx transport results are reported as mean values±range (error bars) from two separate experiments for *wt*hRFC$^{Myc-his10}$/R5 cells (**A**, upper panel) and for *dg*hRFC$^{Myc-his10}$/R5 cells (**B**, upper panel). A representative Western blot is shown for *wt*hRFC$^{Myc-his10}$/R5 cells (**A**, lower panel) and for *dg*hRFC$^{Myc-his10}$/R5 cells (**B**, lower panel). β-actin was used as a loading control. The molecular mass markers for SDS–PAGE are noted.

Received 25 April 2014/28 May 2014; accepted 19 June 2014

Published as Immediate Publication 20 June 2014, doi 10.1042/BSR20140065

© 2014 The Author(s) This is an Open Access article distributed under the terms of the Creative Commons Attribution Licence (CC-BY) (http://creativecommons.org/licenses/by/3.0/) which permits unrestricted use, distribution and reproduction in any medium, provided the original work is properly cited.
